# Supplementary material for: Global pathogenomic analysis identifies known and candidate genetic antimicrobial resistance determinants in twelve species
Source: Nat Commun. 2023 Nov 24;14:7690. doi: 10.1038/s41467-023-43549-9 (PMC10673929; doi:10.1038/s41467-023-43549-9)
Supplement: Supplementary file 1 — Supplementary Information [file 41467_2023_43549_MOESM1_ESM.pdf]

Global pathogenomic analysis identifies known and candidate genetic antimicrobial resistance determinants in twelve species

Jason C. Hyun[1], Jonathan M. Monk[2], Richard Szubin[2], Ying Hefner[2], and Bernhard O. Palsson[1,2,3,4,5]*

[1] Bioinformatics and Systems Biology Program, University of California, San Diego, La Jolla, CA, USA
[2] Department of Bioengineering, University of California, San Diego, La Jolla, CA, USA
[3] Department of Pediatrics, University of California, San Diego, La Jolla, CA, USA
[4] Center for Microbiome Innovation, University of California, San Diego, La Jolla, CA, USA
[5] Novo Nordisk Foundation Center for Biosustainability, Technical University of Denmark, Kemitorvet, Building 220, 2800 Kongens, Lyngby, Denmark
* Corresponding author
Email: palsson@ucsd.edu (BOP)

Supplementary Analysis, Figures, Tables, and References

Supplementary Analysis

Diversity of selected genomes

The overall diversity of the genomes selected for this study was evaluated by examining the distribution of MLST subtypes (from https://github.com/tseemann/mlst) and BioProject IDs (from PATRIC) for each genome, and the number of susceptible/resistant genomes per drug (Supplementary Figure 1, Supplementary Data 2). MLST distributions suggest that the genome collection for each species is genetically diverse, with the most common subtype per species never exceeding 50% of all genomes and at least 44 subtypes represented per species. BioProject ID distributions suggest that a majority of our genome collections also represent a wide range of studies, though the smallest collections were dominated by individual studies (*C. coli* and *C. jejuni* genomes originated almost entirely from PRJNA292668, *E. cloacae* genomes were majority from PRJEB5065) and genomes for *E. faecium* and *N. gonorrhoeae* had poor coverage regarding study of origin. Finally, genome counts by resistance suggest that both susceptible and resistant strains across a wide range of drug classes are present for most species, with only the *N. gonorrhoeae* collection potentially being limited due to AMR data being available for only two drugs.

Observation of TEM-family beta-lactamases in both gram-positive and gram-negative strains

Given the prevalence of TEM family beta-lactamases (*blaTEMs*) among the few AMR genes spanning multiple phylogenetic classes, all observed *blaTEM* alleles were mapped to known *blaTEM* alleles based on RGI annotations, focusing on the 51 complete alleles defined as those with length within 5% of the TEM-1 allele. Complete *blaTEMs* were observed in 4,861 genomes spanning 8 species and were dominated by the TEM-1 allele occurring in 4,424

genomes (Supplementary Figure 4a, Supplementary Table 3, Supplementary Data 4). All but five alleles were within two mutations of TEM-1, and only 9 alleles (including TEM-1) were observed in at least 10 genomes. Individual alleles were largely specific to phylogenetic class with 38 alleles limited to Gammaproteobacteria and 10 alleles limited to *N. gonorrhoeae*, compared to two alleles observed in both (TEM-1 and TEM-135). Just one allele was observed in gram-positive strains, TEM-116 (substitutions V82I and A182V relative to TEM-1), occurring in 11 *S. aureus* strains and 17 *S. enterica* strains. Contigs from genome assemblies harboring TEM-116 were mapped to known plasmids on PLSDB[1] using MASH[2], and the 28 instances of TEM-116 were predicted to be located on one of three plasmids: NZ_AJ437107.1 (14 *S. enterica*, 1 *S. aureus*), NZ_AJ438270.1 (3. *S. enterica*), and NC_019053.1 (10 *S. aureus*) (Supplementary Figure 4b-c, Supplementary Data 4).

Pairwise MASH distances between these genomes were compared to those between all *S. aureus* and *S. enterica* genomes to assess sequence similarity and clonality (Supplementary Figure 5). The 17 *S. enterica* genomes with TEM-116 were highly similar with a median pairwise MASH distance of 0.0004 compared to a median of 0.0153 between all *S. enterica* genome pairs, consistent with the source study confirming all such genomes to be derived from a single clonal lineage of serotype Kentucky ST198, albeit from geographically diverse locations[3]. However, as the presence of the two TEM-116 plasmids in these genomes do not closely track their phylogeny, it is ambiguous as to whether the observed plasmid distribution was established earlier in the lineage and resulted from clonal dissemination, or was a result of more recent horizontal gene transfer events.

In contrast, the 11 *S. aureus* strains with TEM-116 were genetically diverse with a median pairwise MASH distance of 0.0080 compared to a median of 0.0151 between all *S. aureus* genome pairs. The genome 1280.16776, which is predicted to carry plasmid NZ_AJ437107.1 shared by some *S. enterica* genomes, is genetically distinct from the other TEM-116 carrying *S. aureus* genomes. Given the geographic prevalence of the *S. enterica* genomes carrying NZ_AJ437107.1 (annotated as isolated across 10 countries: Djibouti, Egypt, France, Indonesia, Israel, Kenya, Kuwait, Morocco, Tanzania, Vietnam)[3], it is possible that the strain corresponding to 1280.16776 could have acquired this plasmid via horizontal gene transfer from a gram-negative species.

To assess the impact of *blaTEM* on beta-lactam resistance in *S. aureus*, the presence of *blaTEM* and other beta-lactam AMR genes was compared to cefoxitin MIC data, which was available for *S. aureus* genomes harboring *blaTEM*. While only four genomes with *blaTEM* also had MIC data, those genomes (which also carried *mecA* and *blaZ*) had significantly elevated MICs compared to the 29 genomes with just *mecA* and *blaZ* or *mecA* alone (p=0.0074, two-sided Mann-Whitney U-test, n_1=4, n_2=29), suggesting *blaTEM* may confer additional protection from beta-lactams in already resistant *S. aureus* (Supplementary Figure 4c, Supplementary Figure 6). These four strains were isolated from a study of the pork production chain in Shandong, China (BioProject: PRJNA433074)[4], reflecting a geographically confined but genetically diverse sample set. The strains span two MLST subtypes (ST9, ST59), with three isolated from human workers (PATRIC IDs: 1280.16838, 1280.16865, 1280.16862) and one

from a pig (1280.16776).

Association between features annotated as known AMR genes and observed resistance

For each known AMR feature across the 127 species-drug cases examined using the SVM workflow, the set of genomes carrying the feature was identified and the fraction of such genomes that are resistant to the corresponding drug was computed. The distribution of these resistance fractions was computed for each species-drug case individually and in aggregate, combining all known AMR features across all cases (Supplementary Figure 14). The distributions generally concentrated around resistance fractions of 0 or 1, which was quantified by computing the mean of resistance fractions for values either less than or greater than 0.5, for each species-drug case (Supplementary Figure 14b-c). These results suggest that features annotated as known AMR features are more likely than not to be related to resistance and are correctly labeled, i.e. features with resistance fractions closer to 1 are more likely to be resistance determinants, while those with fractions closer to 0 are more likely to be markers for susceptibility such as known fluoroquinolone-susceptible *gyrA* alleles.

Design of the GWAS score for biological relevance

The “GWAS score” was inspired by knowledge of follow-up analyses from past association studies. When hundreds of significant hits are observed, many GWAS and ML studies of AMR will focus on the top X features by effect size (i.e. top 3, 5, 10, 20)[5-7]. The GWAS score mimics this diminishing attention with respect to rank by giving exponentially diminishing marginal GWAS score per recovered known gene as rank increases, specifically, to diminish by half every 10 ranks.

Support vector machine hyperparameter assessment on test cases

Ten test species-drug cases were selected among those with substantial data (at least 1000 SIRs and 50 known AMR genes), with balanced representation by drug class and species phylogenetic class (Supplementary Table 4). Across these 10 cases and 256 hyperparameter (HP) combinations, we find that ensemble size had little impact on either accuracy or biological relevance, while the other HPs significantly impacted both metrics: SVM regularization term C, fraction of samples per estimator, and fraction of features per estimator (Kruskal-Wallis test, $n=256$, $FWER < 0.05$, Bonferroni correction, $m=80$ tests) (Supplementary Figure 7, Supplementary Figure 8a, Supplementary Table 5). More aggressive feature subsampling (smaller fraction of features per estimator) also consistently improved on biological relevance without compromising accuracy (Supplementary Figure 8b). Applying two-sided Mann-Whitney U-tests to test for differences in GWAS scores from using feature fraction 25% over 50%, 50% over 75%, and 75% over 100% yielded p-values of $3.0*10^{-17}$, $6.0*10^{-38}$, and $2.6*10^{-29}$, respectively. Analogous tests for differences in test set MCC yielded $1.5*10^{-7}$, 0.03, and 0.27,

respectively, of which only the test between 25% and 50% is significant (n_1=n_2=64, FWER < 0.05, Bonferroni correction, m=6 tests). Finally, the smallest subset of HP combinations containing nearly-optimal models (within 90% of maximum MCC and GWAS scores) was identified for more efficient HP optimization of the remaining species-drug cases (Supplementary Table 6).

The impact of reducing the number of tested HP combinations was examined as follows. For each of the 10 test species-drug cases, the test set MCCs and GWAS scores across all folds (from 5-fold cross validation) were combined across all models resulting from tested HP combinations, then split into those associated with included HP combinations (n_1=72 HP combinations * 5 folds = 360) and those associated with excluded combinations (n_2=184 HP combinations * 5 folds = 920). Two-sided Mann-Whitney U-tests were applied to determine whether the MCCs or GWAS scores differed significantly between models with included or excluded HP combinations (Supplementary Table 7). Significant differences were observed in 2/10 cases for MCCs and 5/10 cases for GWAS scores (n_1=360, n_2=920, FWER < 0.05, Bonferroni correction, m=20 tests). In 2/2 significant MCC cases and 3/5 significant GWAS score cases, the subset had an equal or higher median score than the excluded combinations, suggesting that the reduction in the set of tested HP combinations only rarely reduces the maximum performance attainable.

Variations on the preliminary feature filter

Variations to the approach of pre-filtering features by log odds ratio (LOR) were evaluated for their impact on downstream model performance. The existing filter of taking the top 50,000 features by LOR (specifically, the features with the 25,000 highest and lowest LORs) was compared to analogous filters taking the top 20,000, 10,000, or 5,000 features by LOR. These were also compared to filters taking the top 50,000, 20,000, 10,000 or 5,000 features by Fisher's exact test p-value when testing for enrichment in resistant genomes, for a total of eight possible feature filters. After applying each filter, SVM ensembles were trained to predict AMR phenotype across the 10 test species-drug cases and 256 HP combinations described in the previous section. The maximum and median test set MCC (from 5-fold cross validation) and GWAS score was computed for each species-drug case and under each filter (Supplementary Figure 15, Supplementary Data 5).

In most of the tested species-drug cases, the maximum and median MCC and GWAS scores are robust to the number of features provided, with a few exceptions under Fisher's exact test filters: Model MCCs for the *E. coli*-gentamicin and *S. aureus*-erythromycin cases and GWAS scores for the *A. baumannii*-amikacin, *S. aureus*-ciprofloxacin, and *S. aureus*-erythromycin cases increase with the number of features. When comparing equal size feature sets generated from filtering by either LOR or Fisher's exact test p-value, models derived from the LOR filter consistently performed better. These results suggest that 1) future applications of this ML workflow may be able to apply stricter preliminary feature filters to reduce the computational resources required for training with relatively little impact on performance, and 2)

the LOR appears to more effectively identify features required for better performing models than the Fisher's exact test p-value, possibly due to its emphasis on effect size over significance.

Generalizability of the GWAS score

To assess whether a model's GWAS score calculated from a fixed set of known AMR genes is representative of its capacity to recover other unseen AMR genes, the following experiment was conducted. For a given species-drug case, 1) half of all known AMR genes were randomly hidden, 2) GWAS scores were computed using either visible or hidden AMR genes across all 256 tested hyperparameter combinations, and 3) the Spearman correlation was computed between the two GWAS scores as a measure of generalizability. This experiment was conducted for n=100 random selections of hidden AMR genes for each of the 10 test species-drug cases (Supplementary Data 5). Overall, the results suggest that the GWAS score often generalizes well to hidden AMR genes, with a median Spearman correlation between GWAS scores from visible vs. hidden AMR genes exceeding 0.4 in 6/10 species-drug cases and 0.6 in 4/10 cases (Supplementary Figure 9a). The extent of generalizability was dependent on the initial level of GWAS score variation, i.e. the standard deviation of GWAS scores across all hyperparameter combinations with all AMR genes visible (Supplementary Figure 9b). Low initial variation in GWAS scores results in a less robust ranking of models by GWAS score, and consequently weaker correlations between GWAS scores calculated using different subsets of known AMR genes. Conversely, cases of high initial variation in GWAS score, i.e. those where HP optimization can meaningfully impact model performance, are more likely to have GWAS scores that are representative of the model's performance at recovering yet unknown AMR genes.

Effect of dataset parameters on model performance

The relationship between six dataset parameters and model performance was analyzed: species, drug class, dataset size (number of genomes), extent of class imbalance (minority phenotype fraction), fraction of genomes originally assigned the "intermediate" phenotype, and total number of known AMR genes annotated (Supplementary Figure 10, Supplementary Table 8). Associations between these parameters and either the model's predictive performance (mean test set MCC from 5-fold cross validation) or known AMR gene recovery (number of AMR genes recovered among the top 20 features) were examined using Spearman R tests for quantitative parameters and Kruskal-Wallis tests for qualitative parameters, across n=127 species-drug cases. Of these, four tests were significant (FWER < 0.05, Bonferroni correction, m=12 tests): Number of genomes vs. MCC, species vs. MCC, fraction of intermediate genomes vs. AMR gene recovery, and total known AMR genes vs. AMR gene recovery.

The significant result regarding intermediate genomes points to an opportunity to improve the overall workflow. Given the negative association between intermediate genomes and model performance (likely due to their genetic similarity to both susceptible and resistant

genomes), future iterations of this workflow may benefit from treating intermediate genomes as a separate phenotype, and original SIR and MIC phenotypes are available in Supplementary Data 2. Nonetheless, the prevalence of intermediate genomes and their potential confounding effects is unlikely to have a significant impact on the final set of AMR gene candidates, as the accurate models (those with MCC > 0.8) used for identifying candidates had a median intermediate genome fraction of only 0.8%. Finally, we note the weak but negative correlation between the number of known AMR genes and model MCC (Spearman correlation = -0.215). This result is consistent with previous ML studies of AMR suggesting that models that accurately predict AMR phenotype often do not rely on known AMR genes and highlights the importance of recognizing phenotype prediction accuracy and recovery of true AMR genes as distinct objectives.

Characterization of gyrA alleles associated with fluoroquinolone resistance recovered using support vector machine ensembles

All *gyrA* alleles among the top 50 features from models related to fluoroquinolone resistance were identified, totaling 30 alleles across 7 species. Mutations were called relative to the wildtype allele of the corresponding species, defined as the most commonly observed *gyrA* allele among genomes susceptible to at least one fluoroquinolone. Resulting mutations and GenBank accession IDs for wildtype alleles are available in Supplementary Data 5. All recovered *gyrA* alleles with positive feature weight for resistance had at least one known resistance-conferring mutation, covering the following substitutions: S81L in *A. baumannii*[8], T86I in *C. coli* and *C. jejuni*[9], S83L and D87N in *E. coli*[10], T83I in *P. aeruginosa*[11], and S83Y and D87* in *S. enterica*[12]. All recovered *gyrA* alleles with negative feature weight for resistance had no such known resistance-conferring mutations. These results suggest that the *gyrA* alleles recovered by the SVM ensemble approach are consistent with current understanding of the *gyrA* mutational landscape with respect to fluoroquinolone resistance.

Selection of AMR gene candidates for experimental validation

AMR gene candidates were identified through a series of filters applied to the highest weighted features in the most accurate AMR models (Figure 4a). The top 10 features by weight (including ties) across the 78 species-drug AMR models achieving test MCC > 0.8 were combined to yield an initial set of 886 unique genetic features predictive of AMR. Of these, 610 were not directly associated with known AMR genes, 519 were also observed in at least 10 genomes, and 347 also had positive log odds ratios (LORs) for resistance against the drug for which the feature was predictive of resistance. Candidates in this intermediate set were then scored based on the sum of the following: 1) number of drugs in the same drug class for which the feature is significantly associated with resistance (applying Fisher's exact test to SIR data or applying Brunner-Munzel test to MIC data), and 2) number of drugs in the same drug class for which the feature appears in at least one resistant genome without any other known AMR genes

(i.e. number of drugs for which this feature could explain previously unexplainable resistance). Finally, for each species-drug class pair, the top 10 features by this score were selected to yield the 142 AMR gene candidates (some pairs had fewer than 10 features remaining after these filters). Results of the scoring process are detailed in Supplementary Data 7.

In the selection of features for experimental validation, the 142 candidates were further categorized by function and filtered down to 43 features that were 1) not poorly characterized, 2) not associated with mobile elements (transposases, insertion elements, phage elements, integrases, plasmid maintenance), 3) not AMR genes for unrelated drugs, and 4) not strongly correlated with a known AMR gene after manual inspection (Figure 4b). Details regarding these 43 features are in Supplementary Data 7. Of these, six features referred to specific sequence variants in *E. coli,* the species the authors were best equipped to test experimentally. Two were excluded in order to focus on allele-type features and take advantage of the Keio knockout collection[13], and ultimately *frdD* and *cycA* were selected for experimental validation for their better functional and metabolic characterization compared to the other two options, *sugE* and *yjfN*.

Distribution of cycA, frdD, and ampC across E. coli genomes

Analysis of all 3,856 *E. coli* genomes in this study suggests that *cycA*, *frdD*, and *ampC* are core genes of *E. coli*, found in 3,823 (99.1%), 3,796 (98.4%), and 3,775 (97.8%) of genomes, respectively. No genomes had multiple copies of *cycA*, *frdD*, or *ampC*. 3,748 (97.2%) genomes had both *frdD* and *ampC*, of which all but three harbored *frdD* and *ampC* on the same contig. The distance between the *frdD* and *ampC* ORFs was highly consistent with a mean of 62.9bp, standard deviation of 5.2bp, and range of 30-192bp. A vast majority of genomes (3,698) had a *frdD*-*ampC* distance of exactly 63bp. This result suggests that a *frdD* V111D mutation will impact the *ampC* promoter in most *E. coli* strains. All instances of *cycA*, *frdD*, and *ampC* identified across all *E. coli* genomes are available in Supplementary Data 8.

Supplementary Figures

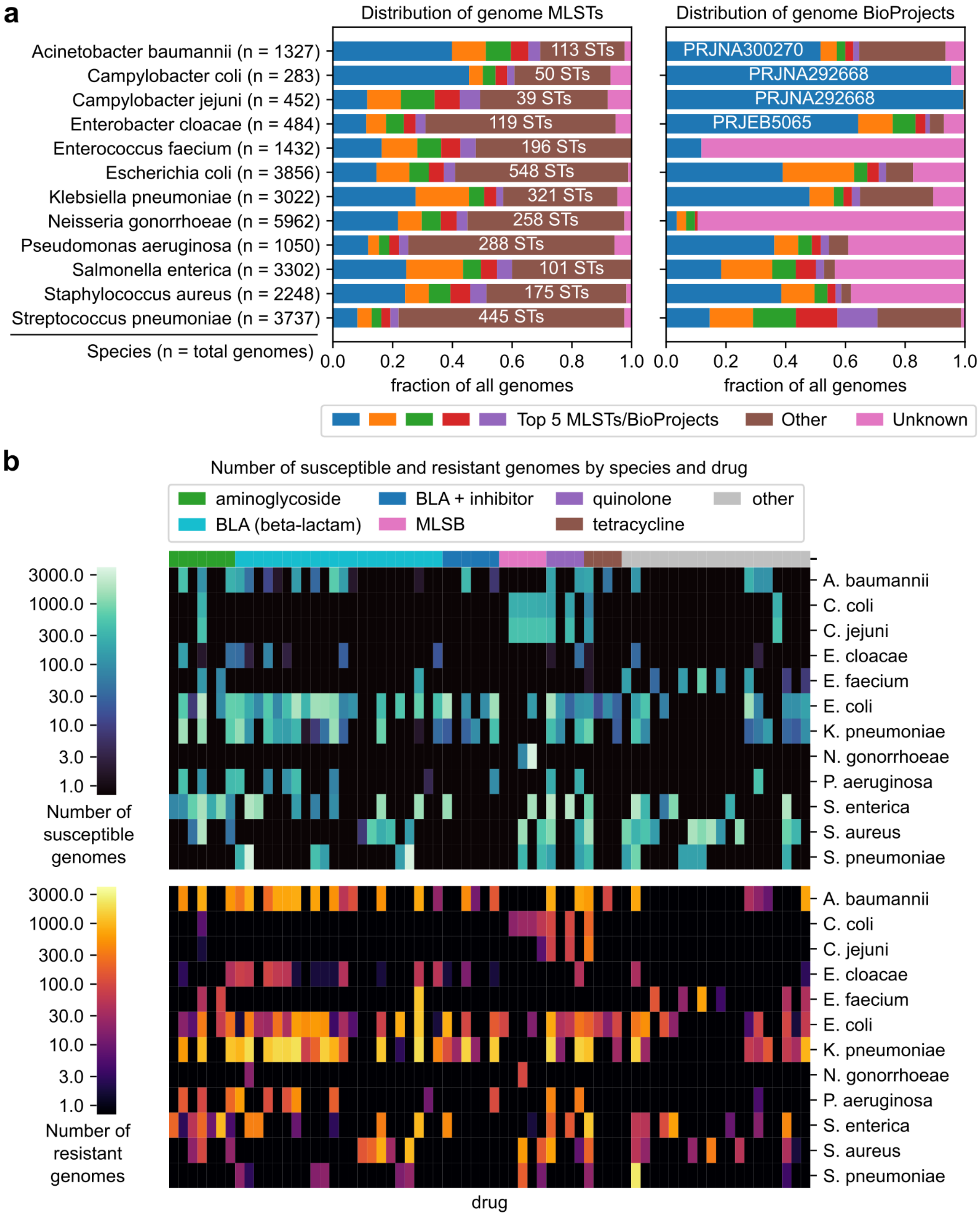

Supplementary Figure 1: Diversity of samples by subtype, study of origin, and AMR phenotype. a) Distribution of MLST subtypes and BioProject accession IDs for each species' genome collection. For MLSTs, the total number of additional MLST subtypes beyond the five most common are labeled. BioProjects comprising at least 50% of genomes for a given species are labeled. b) Distribution of AMR phenotypes by species, drug, and drug class. Drugs are sorted by drug class. Macrolide-lincosamide-streptogramin B is abbreviated "MLSB".

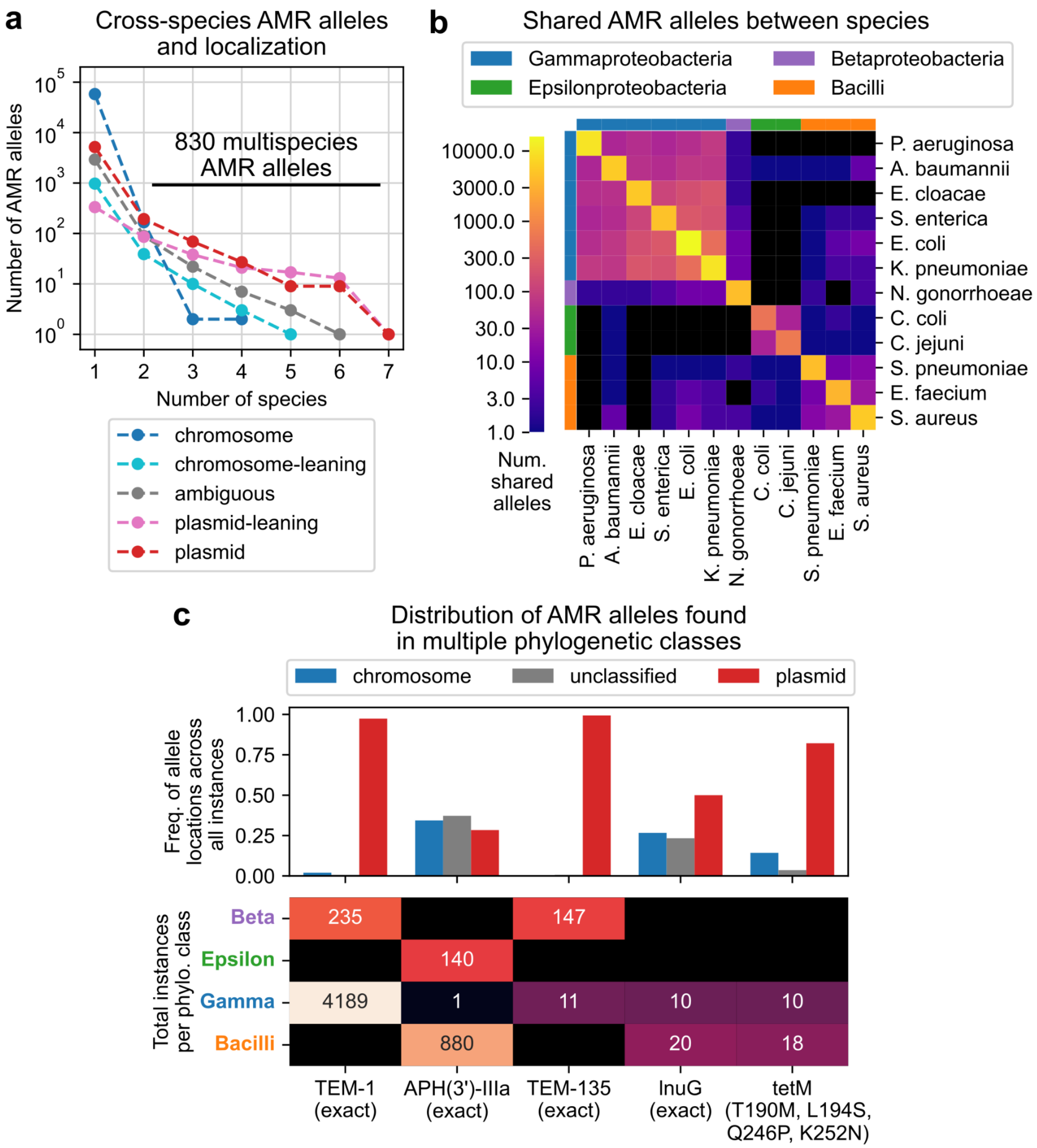

Supplementary Figure 2: Cross-species analysis of 68,324 antimicrobial resistance gene alleles and gene locations. a) Relationship between the number of species an AMR allele is observed in and tendency to be plasmid-encoded. b) Number of AMR alleles shared between each pair of species, compared to species phylogenetic class. c) Distribution of predicted gene locations and total occurrences per phylogenetic class for AMR alleles appearing in at least 10 genomes in multiple classes.

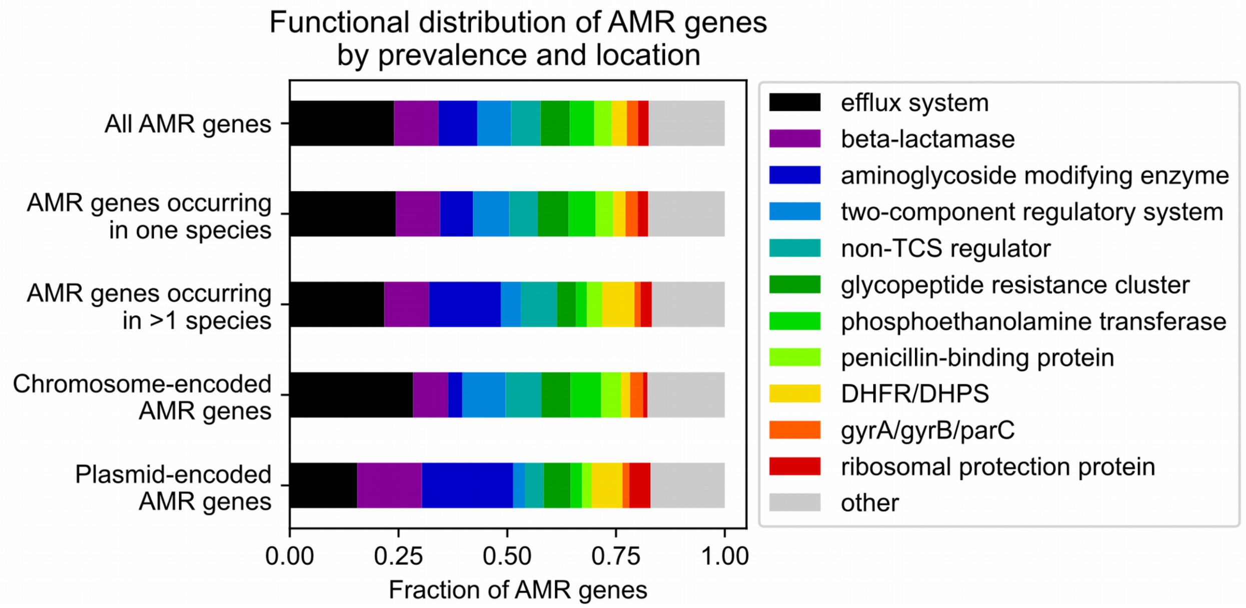

Supplementary Figure 3: Distribution of 6,332 antimicrobial resistance genes across 12 species by functional category, cross-species prevalence, and gene location. Abbreviated functions are two-component regulatory system (TCS), dihydrofolate reductase (DHFR), and dihydropteroate synthase (DHPS).

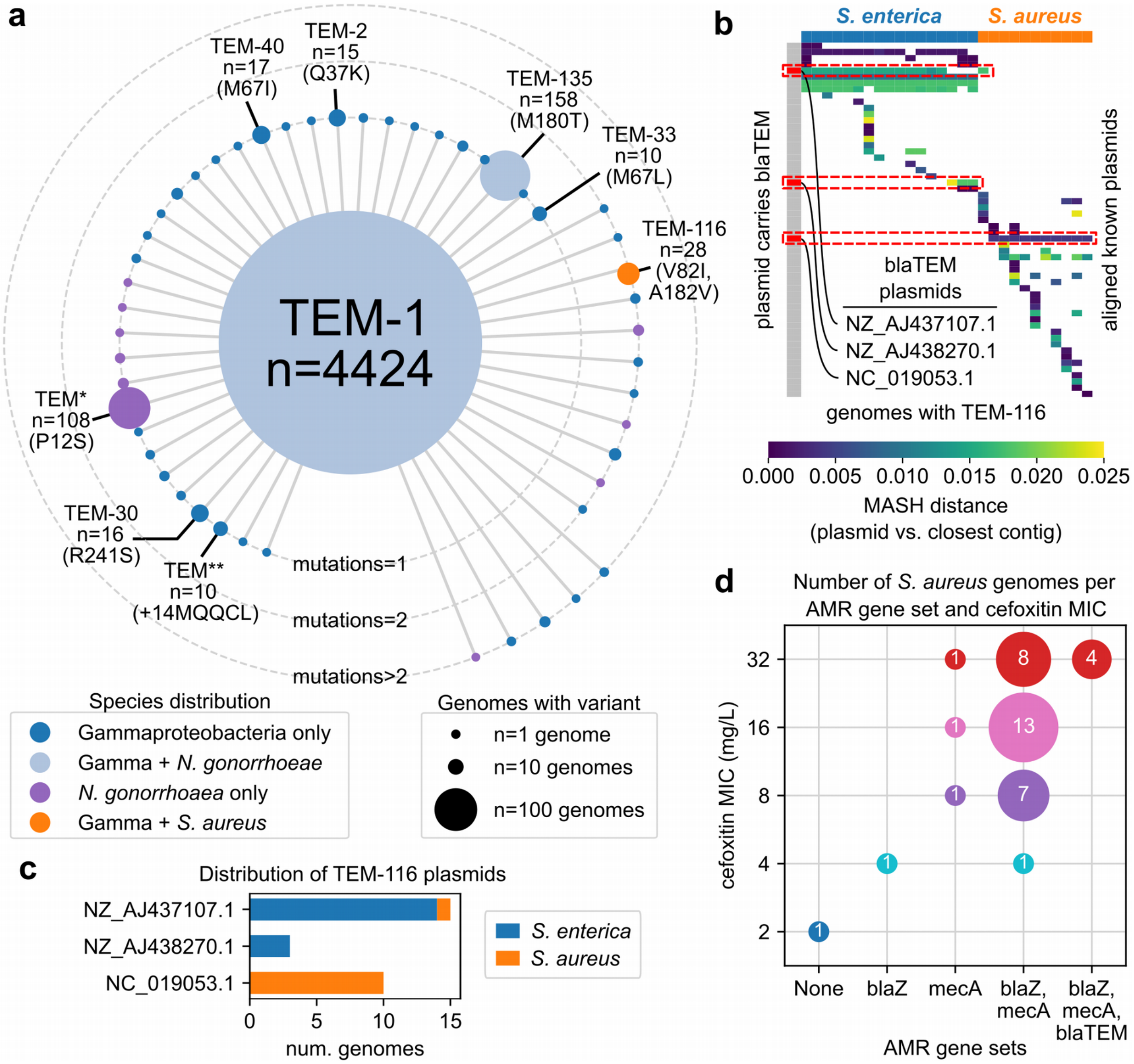

Supplementary Figure 4: Distribution of TEM-family beta-lactamases observed in 4,861 genomes across 8 species. a) Distribution of TEM-family beta-lactamase (*blaTEM*) alleles with respect to genome count, harboring species, and mutations relative to the TEM-1 allele. Alleles occurring in at least 10 genomes are labeled. TEM* and TEM** refer to unnamed *blaTEM* alleles. b) Alignments between contigs from genomes with TEM-116 and PLSDB plasmids based on MASH distance. Known plasmids carrying *blaTEM* are indicated. All plasmids with MASH distance < 0.025 to at least one genome with TEM-116 are shown. c) Distribution of the three plasmids containing TEM-116 by species. d) Relationship between beta-lactam associated AMR genes and cefoxitin minimum inhibitory concentration (MIC) in *S. aureus*. Numeric labels correspond to the number of genomes observed with the corresponding AMR genes and MIC.

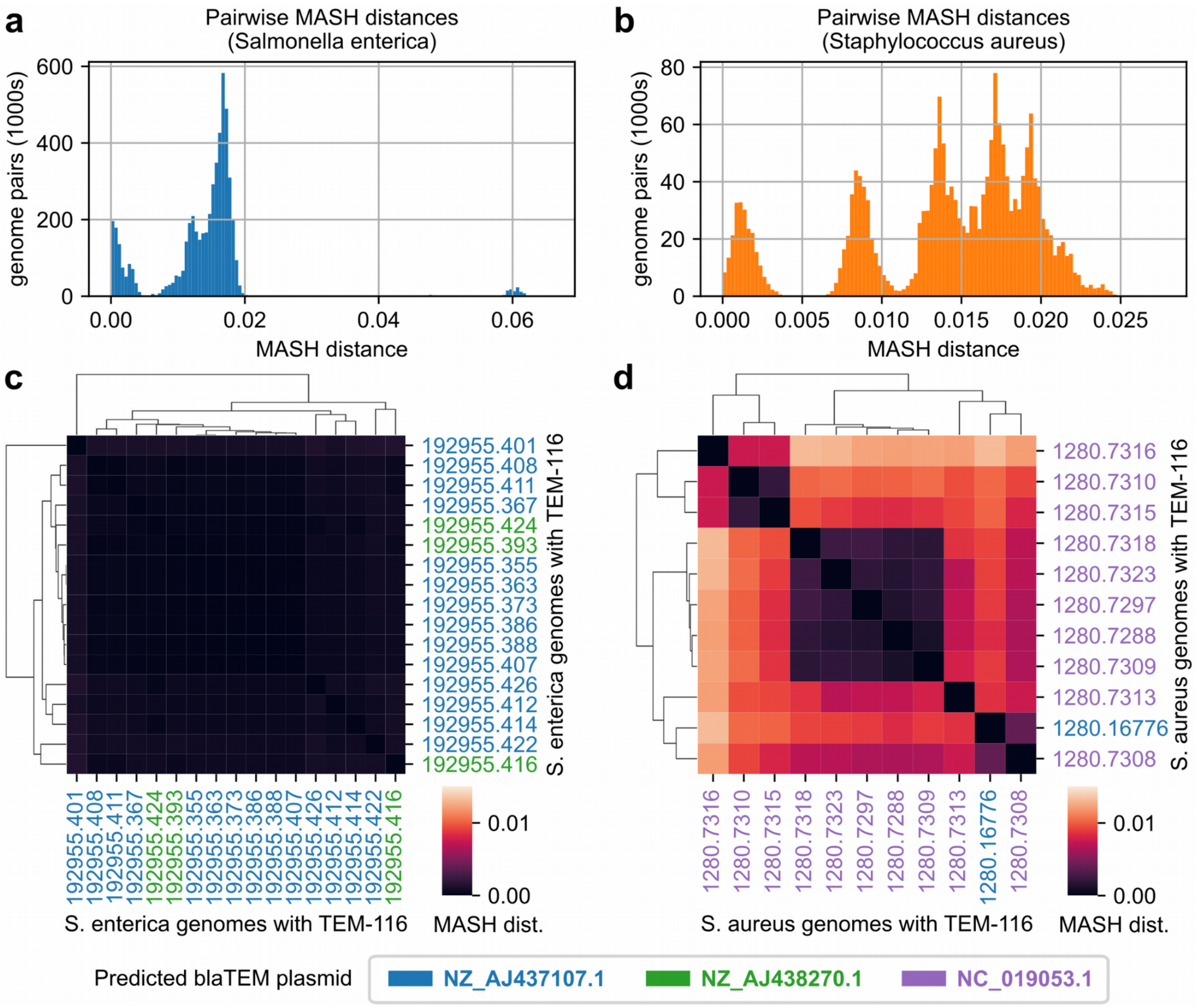

Supplementary Figure 5: Sequence similarity between *S. enterica* and *S. aureus* genomes carrying TEM-116. a-b) Distributions of pairwise MASH distances between all 3,302 *S. enterica* and 2,248 *S. aureus* genomes. c-d) Clustermaps based on pairwise MASH distances between the 17 *S. enterica* and 11 *S. aureus* genomes carrying TEM-116. Heatmaps share the same color scales. Genomes are colored by their predicted *blaTEM* plasmid and are clustered using single linkage and Euclidean distances.

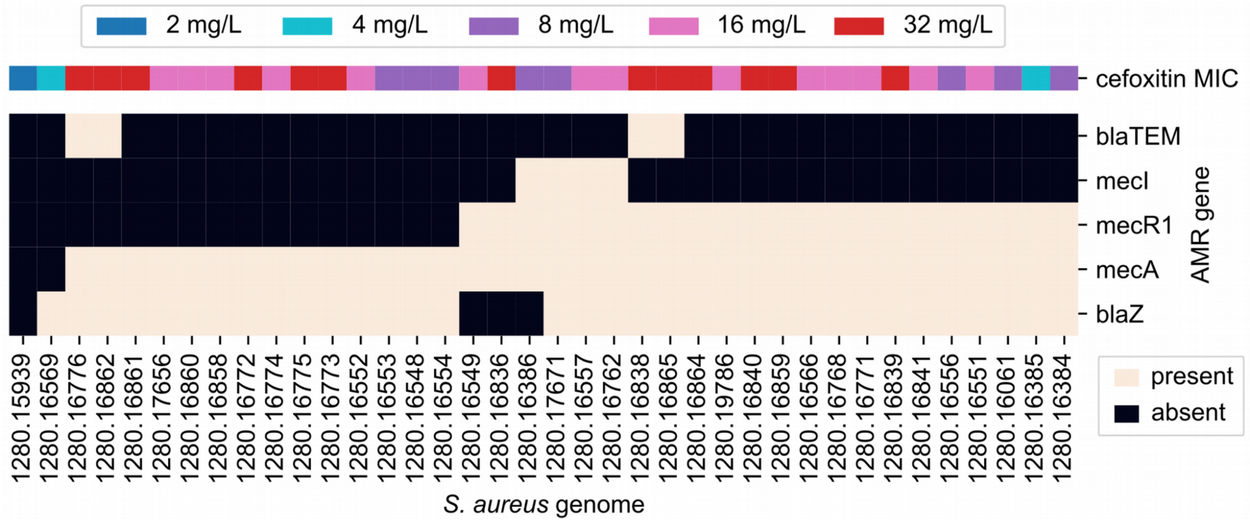

Supplementary Figure 6: Cefoxitin minimum inhibitory concentration (MIC) versus beta-lactam resistance-associated genes across 38 *S. aureus* genomes. Rows and columns have been ordered by hierarchical clustering with Jaccard distances and average linkage.

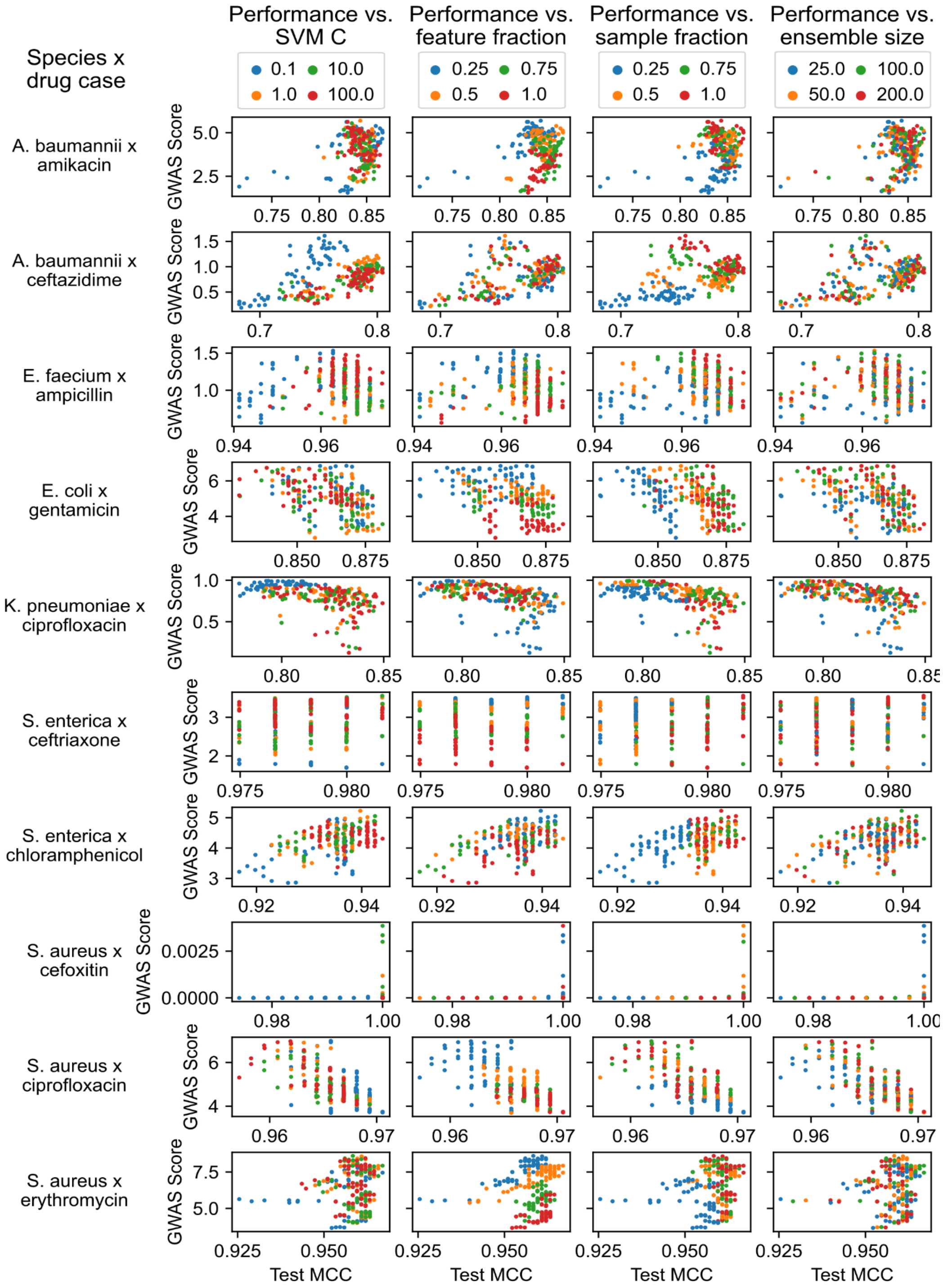

Supplementary Figure 7: Impact of SVM ensemble hyperparameters on AMR phenotype prediction performance and recovery of known AMR genes across 256 hyperparameter combinations and 10 species-drug cases. Each row corresponds to a single AMR prediction problem between a species and a drug, and each column corresponds to a varied hyperparameter. X-axes show average MCCs on the test set from 5-fold cross validation (CV) experiments, y-axes show average GWAS scores across the CV experiment.

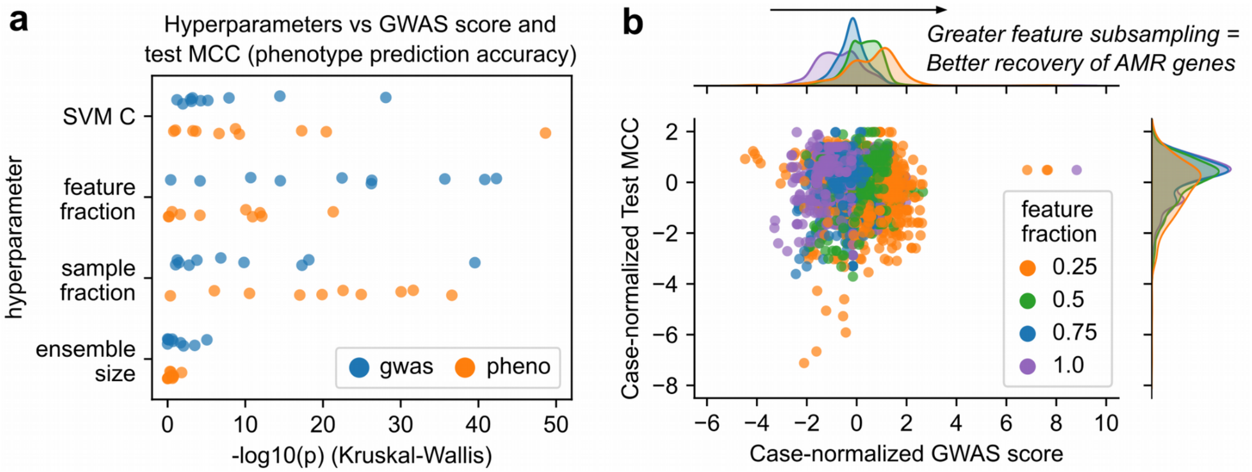

Supplementary Figure 8: Overall impact of SVM ensemble hyperparameters on performance. a) Kruskal-Wallis ANOVA tests between hyperparameters and either AMR phenotype prediction performance (Matthews correlation coefficient; MCC) or biological relevance (GWAS score) across 10 test species-drug cases and n=256 hyperparameter combinations. b) Impact of feature subsampling on model performance for the 10 test cases. MCCs and GWAS scores have been normalized to mean 0 and standard deviation 1, within their specific species-drug cases.

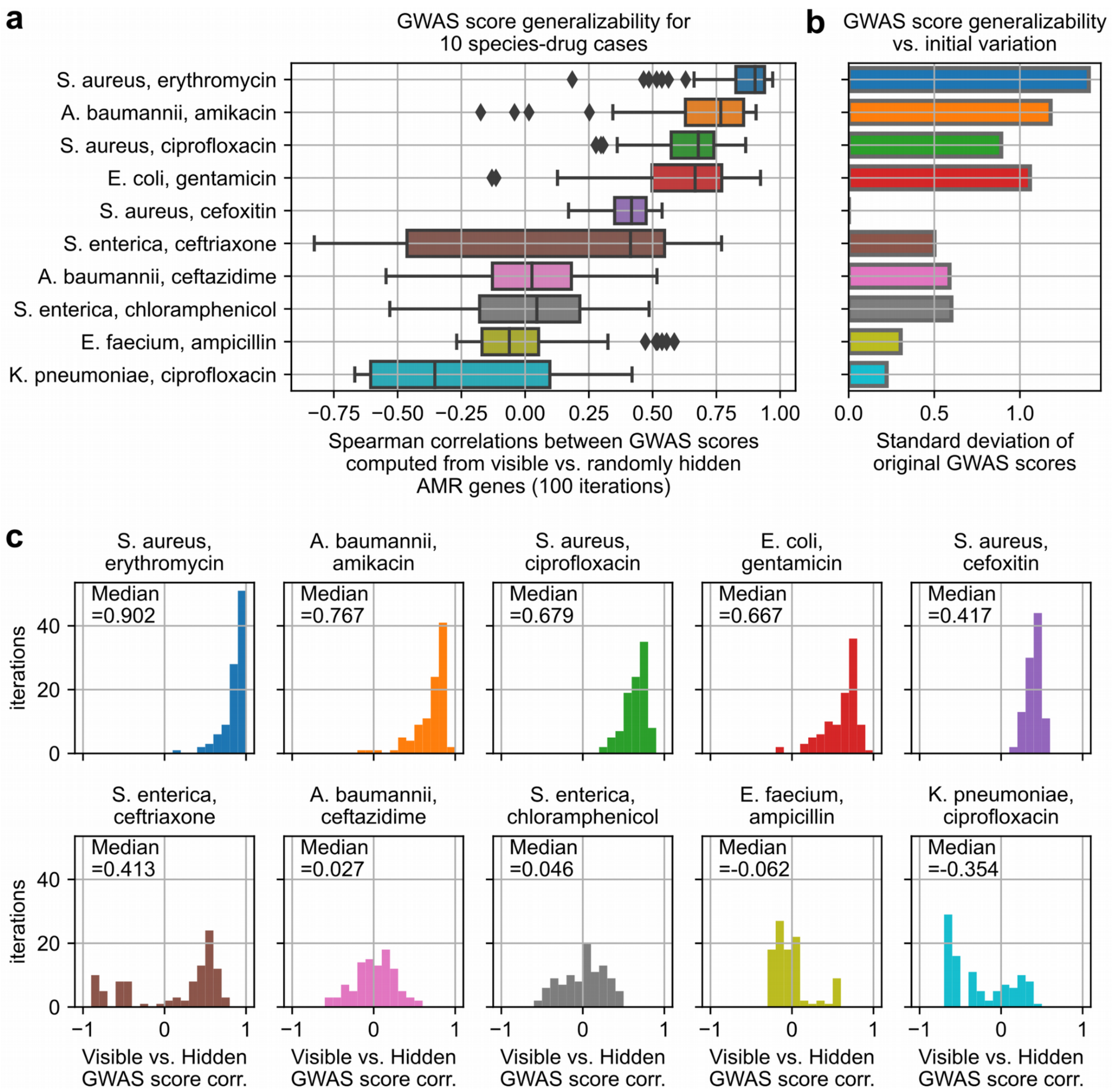

Supplementary Figure 9: Generalizability of the GWAS score. For each species-drug case, half of all known AMR genes were hidden randomly and GWAS scores were computed using either visible or hidden AMR genes for models trained using each possible hyperparameter combination. The distribution of Spearman correlations between the two GWAS scores is shown in (a) for n=100 random selections of hidden AMR genes. Boxplots indicate Q1, median, Q3, whiskers at maximum and minimum values within 1.5*IQR of the median, and outliers outside this range. b) Standard deviation of GWAS scores per species-drug case. c) Distribution of GWAS score Spearman correlations by species-drug case.

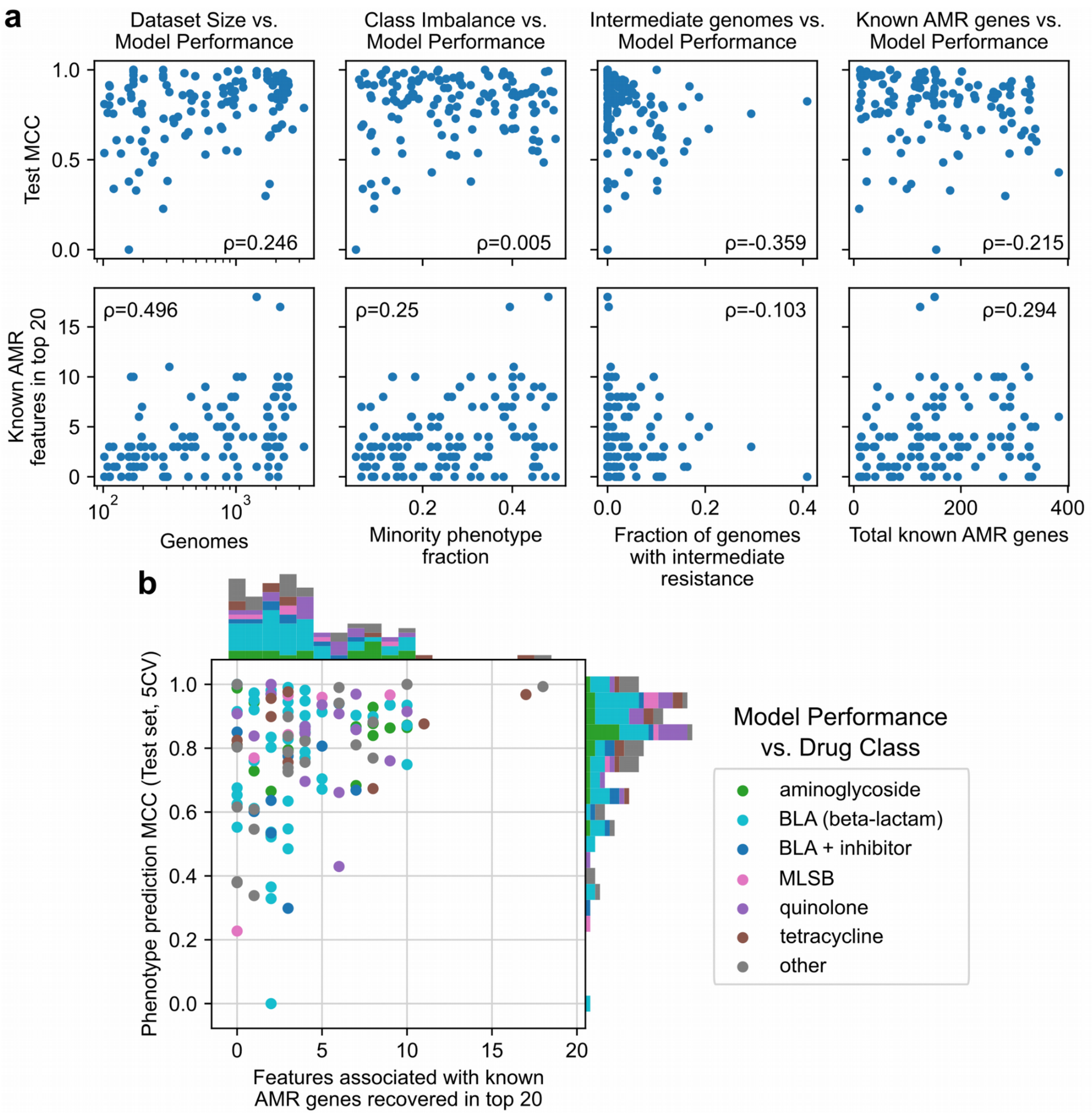

Supplementary Figure 10: Relationship between dataset parameters and model performance across 127 species-drug cases. a) Performance of hyperparameter-optimized SVM ensembles vs. dataset size, extent of class imbalance, abundance of “intermediate” resistant genomes, and total known AMR genes annotated. Spearman correlation coefficients across the n=127 species-drug cases are shown. b) Performance of the 127 SVM ensembles versus drug class.

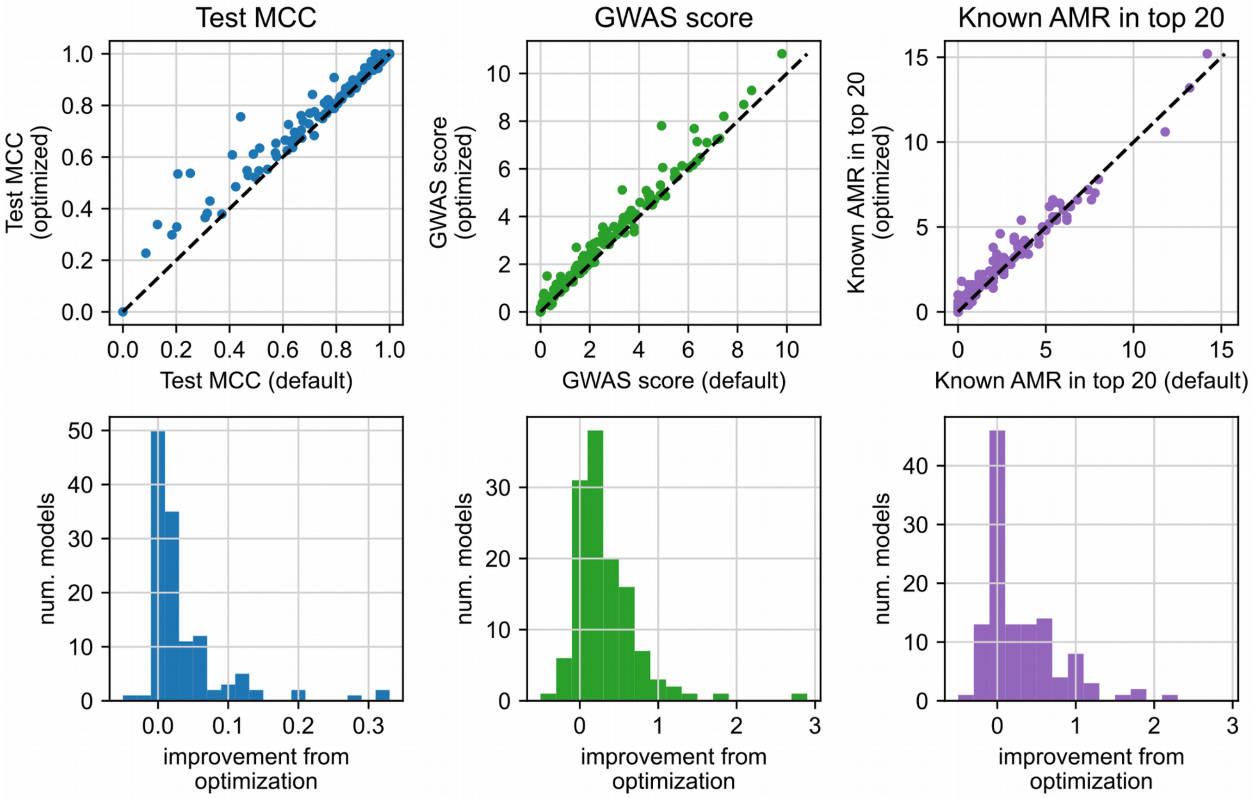

Supplementary Figure 11: Performance improvements from hyperparameter optimization of SVM ensembles across 127 species-drug cases. Performance by three metrics is shown for SVM ensembles trained using default fixed hyperparameters (C = 1, feature fraction = 50%, sample fraction = 75%, ensemble size = 50) compared to ensembles with hyperparameters optimized to balance both phenotype prediction accuracy and known AMR gene recovery. Performance is based on means from 5-fold cross validation.

a

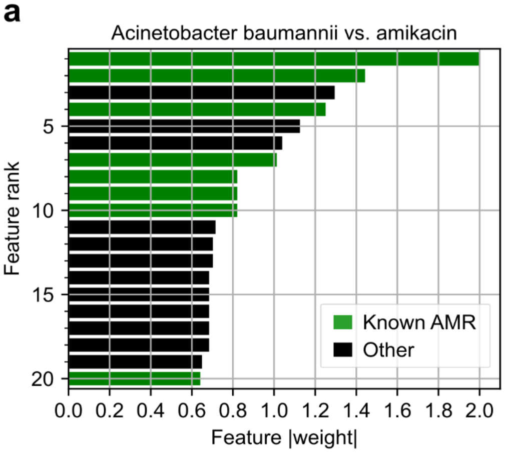

Rank	Found by Pyseer?	Found by Fisher?	Gene	Feature Type
1	X	X	APH(3')-VI	gene
2	-	-	APH(3')-VI	coding_variant
3			hypothetical protein 1	gene
4	X	-	armA	gene
5			hypothetical protein 1	coding_variant
6			hypothetical protein 2	gene
7	-	-	APH(3')-VI	coding_variant
8	-	-	AAC(6')-I	coding_variant
9	-	-	AAC(6')-I	gene
10	X	-	armA	coding_variant
11			hypothetical protein 1	5_variant
12			mobile element 1	coding_variant
13			mobile element 1	gene
14			hypothetical protein 3	gene
15			hypothetical protein 4	coding_variant
16			hypothetical protein 3	coding_variant
17			hypothetical protein 5	3_variant
18			hypothetical protein 5	coding_variant
19			hypothetical protein 1	5_variant
20	X	-	APH(3')-VI	coding_variant

Known AMR Features/Genes: 8/3

b

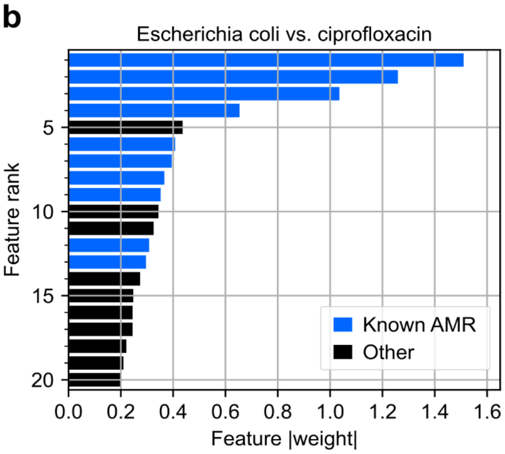

Rank	Found by Pyseer?	Found by Fisher?	Gene	Feature Type
1	X	-	gyrA	coding_variant
2	X	-	parC	coding_variant
3	-	-	gyrA	coding_variant
4	X	X	gyrA	coding_variant
5			5S rRNA	coding variant
6	-	-	gyrA	coding_variant
7	X	-	parE	coding_variant
8	-	-	qnrS	5_variant
9	-	-	gyrA	coding_variant
10			hypothetical protein 6	coding_variant
11			mobile element 2	coding_variant
12	-	-	parC	coding_variant
13	-	-	parC	coding_variant
14			hypothetical protein 7	5_variant
15			yeeV	3_variant
16			eptA	gene
17			eptA	3_variant
18			yihF	5_variant
19			parB	5_variant
20			Mu10 (phage-encoded)	coding_variant

Known AMR Features/Genes: 10/4

c

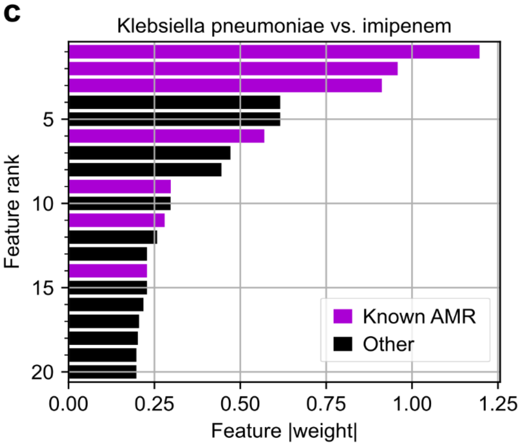

Rank	Found by Pyseer?	Found by Fisher?	Gene	Feature Type
1	X	X	blaKPC	gene
2	X	X	blaKPC	3_variant
3	-	-	blaOXA	gene
4			hypothetical protein 8	coding_variant
5			hypothetical protein 8	gene
6	-	-	blaNDM	gene
7			mobile element 3	gene
8			yeaY	gene
9	-	-	blaOXA	3_variant
10			ompC	gene
11	-	-	blaNDM	coding_variant
12			yafQ	coding_variant
13			hypothetical protein 8	3_variant
14	-	-	blaNDM	3_variant
15			hypothetical protein 8	5_variant
16			srlE	gene
17			hypothetical protein 9	gene
18			ompC	5_variant
19			hypothetical protein 10	coding_variant
20			incW	5_variant

Known AMR Features/Genes: 7/3

Supplementary Figure 12: Visualization of the top 20 genetic features associated with AMR for three species-drug cases as identified by SVM ensembles. Top features are shown for cases a) *A. baumannii* vs. amikacin, b) *E. coli* vs. ciprofloxacin, and c) *K. pneumoniae* vs. imipenem. Features related to known AMR genes are colored and also labeled by whether they were also recovered by Pyseer and/or Fisher's exact test, and other features are black. Features related to undercharacterized genes are labeled as either "hypothetical protein" or "mobile element", numbered by the order the gene is displayed. The type of genetic variant is also shown for each feature. Additional details are in Supplementary Data 5.

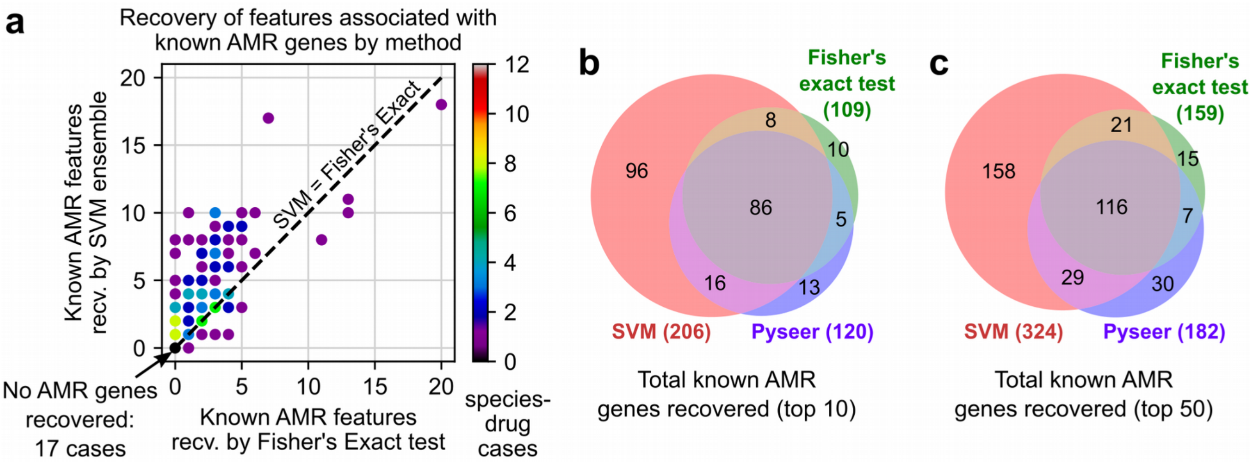

Supplementary Figure 13: Additional comparisons between performance of SVM ensembles, Pyseer, and Fisher's exact test at recovering known AMR genes. a) Comparison between SVMs and Fisher's exact test at recovering known AMR genes across 127 species-drug cases. b-c) Total known AMR gene-drug mappings recovered by SVMs, Pyseer, and Fisher's exact tests across all cases, when defining a recovered gene as those in the top 10 or top 50 features when sorting by feature weight (for SVM) or p-value (for Pyseer and Fisher's exact test).

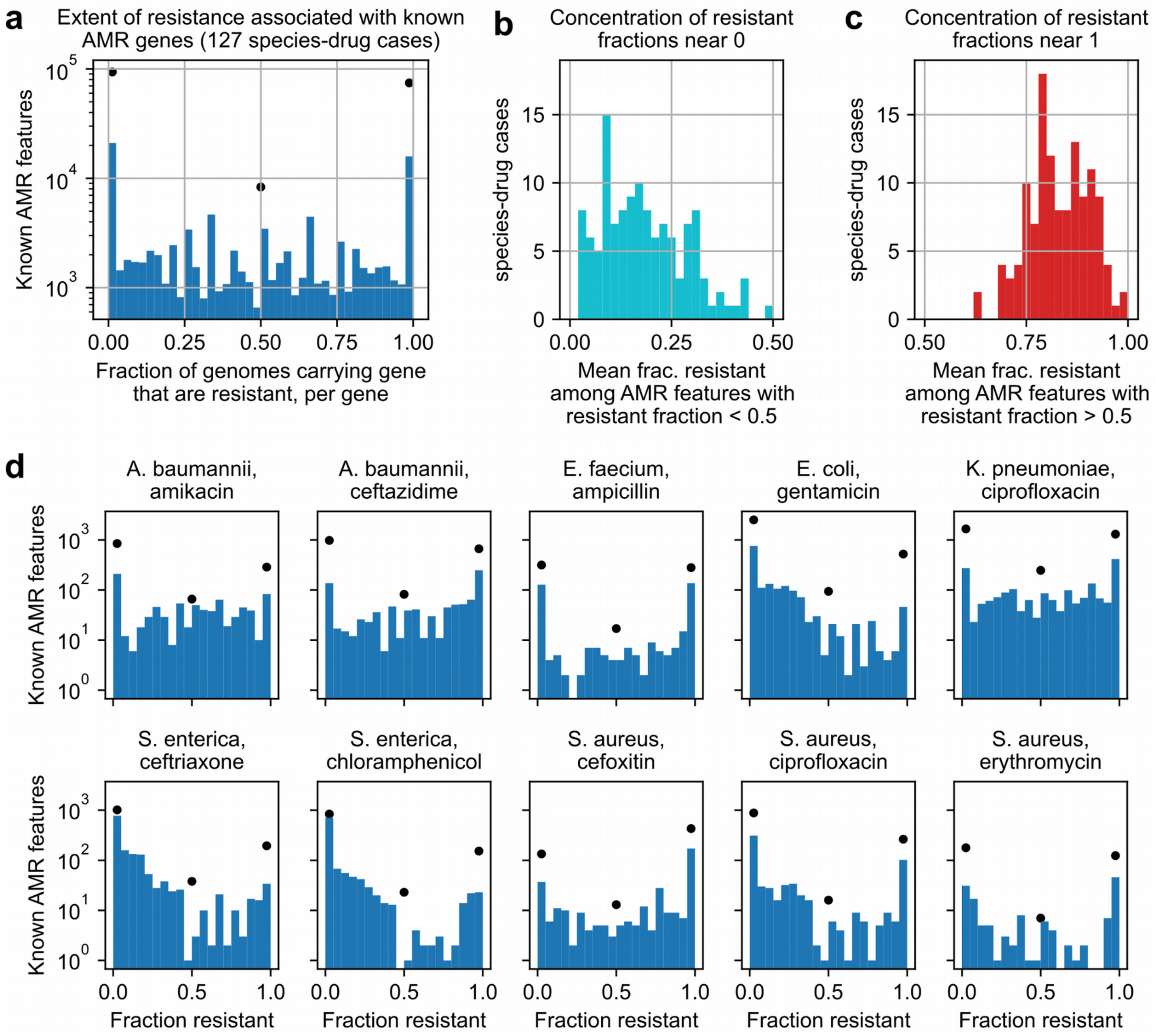

Supplementary Figure 14: Associations between known AMR features and resistance. For each known AMR feature in a given species-drug case, the fraction of genomes with the feature that are resistant to the drug was computed. The distribution of these resistance fractions combined across 127 species-drug cases is shown in (a), with black dots representing rare features (found in no more than 2 genomes) and blue bars representing all other known AMR features. The tendency for these distributions to concentrate near 0 and 1 was quantified by computing means for values b) less than or c) greater than 0.5. Individual distributions for 10 species-drug cases are shown in (d).

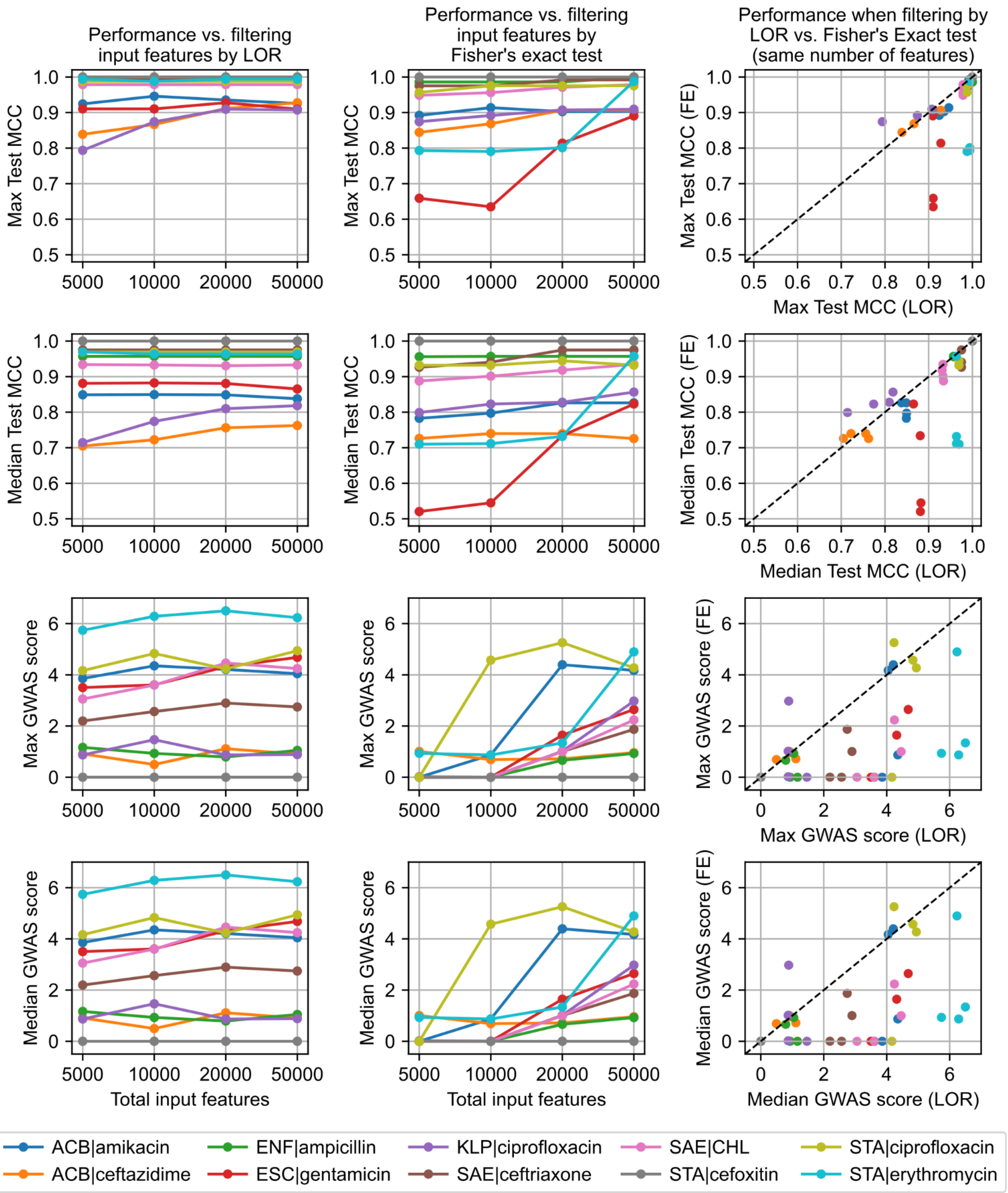

Supplementary Figure 15: Impact of varying the preliminary feature filter on downstream model performance. Each row shows the effect of the feature filter on a model performance metric, either the maximum or median test set MCC (from 5-fold CV) or GWAS score across 256 hyperparameter combinations. The first column shows model performance when limited to the top 5,000, 10,000, 20,000, or 50,000 features after sorting by log odds ratio (LOR) for resistance, and the second column shows model performance under analogous filters sorting instead by Fisher's exact test p-value for resistance. The third column compares performance between LOR and Fisher's exact test filters for identical feature count limits. Each color represents results for a specific species-drug case.

Supplementary Tables

Supplementary Table 1: Genetic feature counts by species.

		ORF-associated				Non-ORF-associated	
species	genomes	genes	alleles	5' flanking variants	3' flanking variants	noncoding clusters	noncoding variants
A. baumannii	1327	43888	239729	262420	275216	177	1725
C. coli	283	8299	44804	32011	31527	77	231
C. jejuni	452	9801	48710	35975	36276	82	381
E. cloacae	484	51969	325374	394577	406576	167	1716
E. faecium	1432	53518	174176	116209	113574	119	890
E. coli	3856	148886	1024333	1005186	1003017	435	5562
K. pneumoniae	3022	94147	562721	584114	596130	356	5010
N. gonorrhoeae	5962	18225	199856	122707	117320	74	693
P. aeruginosa	1050	67217	448882	558844	581936	136	903
S. enterica	3302	56315	262555	255349	261757	322	4932
S. aureus	2248	22649	210749	177169	173244	135	1826
S. pneumoniae	3737	36070	295716	269652	281790	110	1669

Supplementary Table 2: Enrichment of AMR gene categories in multispecies over single species genes and plasmid over chromosomal genes. For each comparison, “genes” is the total number of genes in the category, “LOR” is log2 odds ratio, and “p-value” is two-sided Fisher’s exact test p-value. Starred values are significant (n=6,332 AMR genes, FWER < 0.05, Bonferroni correction, m=36 tests). Categories are sorted by LOR for plasmid over chromosomal genes. Only categories with at least 50 genes were tested.

	**Multispecies over Single species**			**Plasmid over Chromosomal**		
**AMR gene category**	**genes**	**LOR**	**p-value**	**genes**	**LOR**	**p-value**
aminoglycoside modifying enzymes (AMEs)	563	1.272	3.8×10^{-16}*	455	2.957	3.2×10^{-89}*
ribosomal protection proteins	154	0.113	0.72879	115	2.348	4.2×10^{-17}*
chloramphenicol acetyltransferases (CATs)	91	1.001	0.00656	77	1.850	3.4×10^{-8}*
dihydrofolate reductases (DHFRs), dihydropteroate synthases (DHPSs)	219	1.498	1.3×10^{-10}*	191	1.845	1.3×10^{-17}*
rpoB variants	74	-0.718	0.24769	60	1.540	0.00007*
rRNA methyltransferases (rRNA MTases)	152	0.061	0.81658	132	1.049	0.00008*
beta-lactamases	645	0.015	0.90646	541	1.004	2.2×10^{-13}*
glycopeptide resistance clusters (RCs)	425	-0.721	0.00217	357	-0.123	0.50527
gyrA/gyrB/parC variants	165	-1.021	0.01013	141	-0.979	0.00174
non-two-component system (non-TCS) regulators	429	0.358	0.06540	388	-0.993	1.3×10^{-7}*
penicillin-binding proteins (PBPs)	254	-0.257	0.41414	211	-1.088	0.00002*
aminoacyl-tRNA synthetases (AARSs)	101	-1.454	0.01001	82	-1.094	0.00939
efflux	1519	-0.215	0.08753	1346	-1.107	1.7×10^{-24}*
phosphoethanolamine (pEtN) transferases	358	-1.373	1.0×10^{-6}*	316	-1.485	2.1×10^{-11}*
other	410	-0.218	0.34748	362	-1.811	7.4×10^{-17}*
two-component system (TCS) regulators	494	-0.900	0.00005*	429	-1.988	1.6×10^{-22}*
cya variants	57	-0.544	0.45547	50	-3.273	0.00002*
porins	74	-1.595	0.01988	69	-3.762	4.8×10^{-8}*

Supplementary Table 3: Distribution of the most frequently observed TEM-family beta-lactamases by species. Mutations are shown relative to the TEM-1 variant. Variants are ordered by total count. Species are abbreviated as *A. baumannii* (AcB), *E. cloacae* (EnC), *E. coli* (EsC), *K. pneumoniae* (KlP), *N. gonorrhoeae* (NeG), *P. aeruginosa* (PsA), *S. enterica* (SaE), and *S. aureus* (StA).

	Number of genomes with variant by species									
**Mutations**	**Variant**	**AcB**	**EnC**	**EsC**	**KlP**	**NeG**	**PsA**	**SaE**	**StA**	**Total**
-	TEM-1	414	171	1692	1179	235	1	732	-	4424
M180T	TEM-135	-	-	8	1	147	-	2	-	158
P12S	*	-	-	-	-	108	-	-	-	108
V82I, A182V	TEM-116	-	-	-	-	-	-	17	11	28
M67I	TEM-40	-	-	16	1	-	-	-	-	17
R241S	TEM-30	-	-	16	-	-	-	-	-	16
Q37K	TEM-2	-	14	-	1	-	-	-	-	15
M67L	TEM-33	-	-	7	1	-	-	2	-	10
14insMQQCL	*	-	-	-	-	-	-	10	-	10

* No exact match in the CARD database

Supplementary Table 4: Initial test species-drug cases for hyperparameter testing. Columns "n", "% Sus." and "AMR genes" refer to the number of genomes with AMR data, the percent of those genomes that are susceptible, and the number of known AMR genes identified that are associated with the drug for that species, respectively.

Species	Species Class	Drug	Drug Class	n	% Sus.	AMR genes
A. baumannii	γ-proteobacteria	amikacin	aminoglycoside	924	49.1	150
A. baumannii	γ-proteobacteria	ceftazidime	beta-lactam	960	15.3	151
E. coli	γ-proteobacteria	gentamicin	aminoglycoside	2447	86.6	263
K. pneumoniae	γ-proteobacteria	ciprofloxacin	quinolone	2089	19.1	227
S. enterica	γ-proteobacteria	ceftriaxone	beta-lactam	2259	84.2	154
S. enterica	γ-proteobacteria	chloramphenicol	other	2348	82.4	116
E. faecium	Bacilli	ampicillin	beta-lactam	1432	14.2	81
S. aureus	Bacilli	cefoxitin	beta-lactam	1041	23.5	68
S. aureus	Bacilli	ciprofloxacin	quinolone	1725	62.2	42
S. aureus	Bacilli	erythromycin	other	2014	71.7	44

Supplementary Table 5: Negative log10 p-values for Kruskal-Wallis tests between SVM ensemble hyperparameters and model performance. Performance metrics compared are phenotype prediction performance (mean test set MCC during 5-fold cross validation) and recovery of known AMR genes among model features (GWAS score). Each test was conducted across n=256 hyperparameter combinations.

	**Hyperparameter vs. Test set MCC**				**Hyperparameter vs. GWAS score**			
**AMR Case**	SVM C	feature fraction	sample fraction	ensemble size	SVM C	feature fraction	sample fraction	ensemble size
A. baumannii, amikacin	1.009	12.123	6.016	0.75	1.967	26.219	17.297	0.711
A. baumannii, ceftazidime	17.258	0.187	24.916	0.58	3.212	0.402	39.543	0.011
E. coli, gentamicin	3.256	10.935	31.611	0.051	2.963	35.68	1.47	5.058
E. faecium, ampicillin	8.738	11.894	17.011	0.307	7.905	22.447	9.831	1.674
K. pneumoniae, ciprofloxacin	20.421	1.621	19.877	0.051	28.08	4.177	2.767	3.495
S. aureus, cefoxitin	48.629	0.117	0.353	0.001	14.449	10.696	1.244	0.001
S. aureus, ciprofloxacin	9.228	21.314	10.536	0.416	1.211	26.275	18.174	0.059
S. aureus, erythromycin	3.635	10.07	22.577	0.723	3.065	40.817	6.849	0.158
S. enterica, ceftriaxone	0.791	4.16	36.582	0.618	4.264	42.314	1.036	0.59
S. enterica, chloramphenicol	6.62	0.579	30.051	1.804	5.181	14.501	3.774	2.055

Supplementary Table 6: SVM hyperparameter ranges used during optimization. Larger initial ranges were used for evaluating the 10 test species-drug cases, from which reduced ranges were derived for evaluating against all 127 species-drug cases.

**Hyperparameter**	**Initial range**	**Reduced range**
Number of estimators	25, 50, 100, 200	25, 50
Fraction of samples per estimator	25%, 50%, 75%, 100%	50%, 75%, 100%
Fraction of features per estimator	25%, 50%, 75%, 100%	25%, 50%, 75%, 100%
C (SVM regularization term)	0.1, 1, 10, 100	0.1, 1, 10
**Unique combinations**	256	72

Supplementary Table 7: Statistically significant differences in model performance between selected and excluded hyperparameter combinations for 10 species-drug cases. For each case, a two-sided Mann-Whitney U-test was conducted to determine if the trained SVM model's test set MCCs or GWAS scores, across all hyperparameter combinations (HPCs) and folds from 5-fold CV, differed significantly for HPCs included in the reduced HPC range than for excluded HPCs. See Supplementary Table 6 for HPC sets. Starred cases are significant (n_1=72 HPCs * 5 folds = 360, n_2=184 HPCs * 5 folds = 920, FWER < 0.05, Bonferroni correction, m=20 tests).

	**Test set MCC**			**GWAS Score**		
**AMR Case**	Included Median	Excluded Median	MW test p-value	Included Median	Excluded Median	MW test p-value
A. baumannii, amikacin	0.83782	0.83815	0.23016	4.04567	4.07742	0.04913
A. baumannii, ceftazidime	0.76253	0.75629	0.00138*	0.9075	0.59558	$7.1*10^{-11}$*
E. faecium, ampicillin	0.95698	0.95698	0.12555	1.04808	1.07578	0.1873
E. coli, gentamicin	0.86522	0.86143	$2.4*10^{-9}$*	4.68256	5.1806	$1.0*10^{-13}$*
K. pneumoniae, ciprofloxacin	0.81835	0.81573	0.41189	0.88562	0.87238	0.34162
S. enterica, ceftriaxone	0.9751	0.9751	0.04874	2.74439	3.12598	$1.0*10^{-17}$*
S. enterica, chloramphenicol	0.93276	0.93276	0.12058	4.24349	4.28149	0.77218
S. aureus, cefoxitin	1.00000	1.00000	0.94434	0.00000	0.00000	$4.1*10^{-13}$*
S. aureus, ciprofloxacin	0.96917	0.96917	0.60248	4.94068	4.67576	0.00018*
S. aureus, erythromycin	0.96318	0.9575	0.04671	6.23253	6.23332	0.37441

Supplementary Table 8: Impact of input data properties on SVM ensemble performance. Impact was assessed with Kruskal-Wallis tests for categorical properties (species, drug class) and Spearman correlation for quantitative properties (number of genomes, minority phenotype fraction, fraction of genomes with the “intermediate” phenotype, total number of known AMR genes). Spearman correlation tests are two-sided. Each test was conducted over all 127 species-drug cases, and rows are sorted by p-value. Starred cases are significant (n=127, FWER < 0.05, Bonferroni correction, m=12 tests).

Performance Metric	Data Metric	Statistical Test	p-value
AMR genes in top 20	num. genomes	Spearman-R	$3.1*10^{-9}$*
Test MCC in 5CV	species	Kruskal-Wallis	$7.6*10^{-7}$*
Test MCC in 5CV	intermediate fraction	Spearman-R	0.00003*
AMR genes in top 20	total known AMR genes	Spearman-R	0.00079*
AMR genes in top 20	minority fraction	Spearman-R	0.00464
Test MCC in 5CV	num. genomes	Spearman-R	0.00537
AMR genes in top 20	species	Kruskal-Wallis	0.00721
Test MCC in 5CV	total known AMR genes	Spearman-R	0.01536
AMR genes in top 20	intermediate fraction	Spearman-R	0.25039
Test MCC in 5CV	drug class	Kruskal-Wallis	0.30250
AMR genes in top 20	drug class	Kruskal-Wallis	0.48732
Test MCC in 5CV	minority fraction	Spearman-R	0.95993

Supplementary Table 9: Summary of known AMR gene-drug mappings recovered by Fisher's exact test but missed by the SVM ensemble approach. Drugs abbreviated are quinupristin-dalfopristin (Q-D) and trimethoprim-sulfamethoxazole (SXT).

Species	Drug	Missed Gene	Model Test MCC (5CV)	Gene Rank in Model	Statistically Unique Features in Top 50*	Possible Failure Mode
Acinetobacter baumannii	tobramycin	*aadA***	0.87	33	44	Feature rank below top 20 threshold
Enterobacter cloacae	cefepime	*blaKPC***	0.61	-	46	Poor model performance
Enterococcus faecium	Q-D	*eatAv*	1.00	-	1	High correlation among top features
Enterococcus faecium	teicoplanin	*vanXA*	1.00	21	11	Feature rank below top 20 threshold
Enterococcus faecium	teicoplanin	*vanA*	1.00	21	11	Feature rank below top 20 threshold
Enterococcus faecium	teicoplanin	*vanHA*	1.00	21	11	Feature rank below top 20 threshold
Enterococcus faecium	vancomycin	*vanZA***	0.99	-	16	High correlation among top features
Enterococcus faecium	vancomycin	*vanYA***	0.99	-	16	High correlation among top features
Escherichia coli	norfloxacin	*parC*	0.43	-	36	Poor model performance
Klebsiella pneumoniae	SXT	*sul1*	0.84	-	13	High correlation among top features
Neisseria gonorrhoeae	erythromycin	*mtrR*	0.77	-	48	Poor model performance
Staphylococcus aureus	ciprofloxacin	*arlR*	0.97	-	46	-

*Refers to the number of features remaining among the SVM model's top 50 features by weight after collapsing perfectly correlated features together. Lower values correspond to more highly correlated features.

**Also in the top 20 features by Pyseer.

Supplementary Table 10: 13 gene flanking noncoding variants predicted to be associated with resistance against specific drug classes for individual species. Accession IDs are provided for the most common allele of the corresponding gene cluster (RefSeq when possible, GenBank otherwise), along with gene names when available and gene products. Mutations are defined relative to the most common 5'/3' variant for the corresponding gene. The number of resistant/susceptible genomes and log2 odds ratios (LORs) for resistance are shown for the top three drugs by LOR when data for more than three related drugs was available. Drug abbreviations and mappings to relevant sequences are available in Supplementary Data 7.

Species	Drug Class	Accession (Gene)	Predicted Gene Product	Mutations*	Resistant/ Susceptible	LORs
			5' flanking variants			
Acinetobacter baumannii	beta-lactam	AGQ10471.1 (*pqqA*)	Coenzyme PQQ synthesis protein A	-12_2delTGATTTAATCAAGTG**	CTX=73/0<br>CRO=73/0<br>AMP=73/0	CTX=6.4<br>CRO=6.4<br>AMP=6.4
Acinetobacter baumannii	tetracycline	WP_000096554.1 (*clpV*)	T6SS AAA+ chaperone	Most common variant	TET=241/12<br>MIN=71/18	TET=3.3<br>MIN=2.3
Campylobacter coli	quinolone	WP_002805020.1	GNAT acetyl-transferase	Most common variant	CIP=94/71<br>NAL=95/70	CIP=6.0<br>NAL=5.5
Campylobacter coli	quinolone	WP_002783313.1	Putative transmembrane transport protein, MFS	Most common variant	NAL=97/115<br>CIP=95/117	NAL=5.5<br>CIP=5.4
Escherichia coli	beta-lactam	AAG56074.1 (*ymgF*)	Small inner membrane protein	-19A>C, -23A>G, -25_24delTC, -30insA, -48G>A, -173A>T, -175C>A, -211A>T, -213C>T	CTX=28/0<br>CAZ=30/1<br>CXM=14/1	CTX=6.7<br>CAZ=5.7<br>CXM=4.6
Escherichia coli	quinolone	WP_000017703.1 (*hybB*)	Ni/Fe-hydrogenase 2 b-type cytochrome subunit	Most common variant	LVX=260/56<br>CIP=606/606<br>NAL=45/16	LVX=3.3<br>CIP=2.8<br>NAL=1.4
Streptococcus pneumoniae	beta-lactam	WP_000449822.1	Thiaminase II	Most common variant	AMX=13/4<br>MEM=16/1<br>CXM=16/1	AMX=12.4<br>MEM=9.6<br>CXM=9.3
Streptococcus pneumoniae	beta-lactam	ADI69655.1 (*nplT*)	Neopullulanase	Most common variant	AMX=13/3<br>MEM=15/1<br>CXM=15/1	AMX=12.7<br>MEM=9.1<br>CXM=8.6
Streptococcus pneumoniae	beta-lactam	WP_000592948.1 (*ugl*)	Unsaturated chondroitin disaccharide hydrolase	-186C>T	AMX=13/6<br>CXM=17/2<br>MEM=17/3	AMX=11.9<br>CXM=9.6<br>MEM=9.2
			3' flanking variants			
Campylobacter coli	quinolone	WP_002777456.1 (*hspR*)	Transcriptional repressor of DnaK operon	Most common variant	NAL=98/115<br>CIP=96/117	NAL=7.4<br>CIP=7.4

Klebsiella pneumoniae	beta-lactam	WP_000679427.1 (*qacEΔ1*)	Small multidrug resistance (SMR) efflux transporter	Most common variant	AMP=759/0<br>CEF=27/0<br>CRO=772/4	AMP=10.3<br>CEF=5.5<br>CRO=4.3
Salmonella enterica	quinolone	WP_012772747.1	Psp operon transcriptional activator	Most common variant	NAL=9/11<br>CIP=9/10	NAL=3.5<br>CIP=3.2
Streptococcus pneumoniae	beta-lactam	WP_000145597.1 (*nplT*)	Neopullulanase	Most common variant	AMX=13/3<br>MEM=15/1<br>CXM=15/1	AMX=12.7<br>MEM=9.1<br>CXM=8.6

*Mutations are denoted relative to the start codon for 5’ variants. Position -1 corresponds to the first base pair immediately adjacent on the 5’ side of the gene’s start codon.

**Results in the deletion of a GTG start codon and the 12 base pairs immediately adjacent on the 5’ side, with respect to the most common variant. The candidate variant begins with an ATG start codon.

Supplementary References

1. Schmartz, G. P. *et al.* PLSDB: advancing a comprehensive database of bacterial plasmids. *Nucleic Acids Res.* **50**, D273–D278 (2022).

2. Ondov, B. D. *et al.* Mash: fast genome and metagenome distance estimation using MinHash. *Genome Biol.* **17**, 132 (2016).

3. Hawkey, J. *et al.* Global phylogenomics of multidrug-resistant Salmonella enterica serotype Kentucky ST198. *Microb Genom* **5**, (2019).

4. Bi, Z. *et al.* Identical genotypes of community-associated MRSA (ST59) and livestock-associated MRSA (ST9) in humans and pigs in rural China. *Zoonoses Public Health* **65**, 367–371 (2018).

5. Kim, J. *et al.* VAMPr: VAriant Mapping and Prediction of antibiotic resistance via explainable features and machine learning. *PLoS Comput. Biol.* **16**, e1007511 (2020).

6. Nguyen, M. *et al.* Using Machine Learning To Predict Antimicrobial MICs and Associated Genomic Features for Nontyphoidal. *J. Clin. Microbiol.* **57**, (2019).

7. Earle, S. G. *et al.* Identifying lineage effects when controlling for population structure improves power in bacterial association studies. *Nat Microbiol* **1**, 16041 (2016).

8. Vila, J., Ruiz, J., Goñi, P., Marcos, A. & Jimenez de Anta, T. Mutation in the gyrA gene of quinolone-resistant clinical isolates of Acinetobacter baumannii. *Antimicrob. Agents Chemother.* **39**, 1201–1203 (1995).

9. Han, J. *et al.* A fluoroquinolone resistance associated mutation in gyrA Affects DNA supercoiling in Campylobacter jejuni. *Front. Cell. Infect. Microbiol.* **2**, 21 (2012).

10. Weigel, L. M., Steward, C. D. & Tenover, F. C. gyrA mutations associated with fluoroquinolone resistance in eight species of Enterobacteriaceae. *Antimicrob. Agents Chemother.* **42**, 2661–2667 (1998).

11. Takenouchi, T., Sakagawa, E. & Sugawara, M. Detection of gyrA mutations among 335 Pseudomonas aeruginosa strains isolated in Japan and their susceptibilities to fluoroquinolones. *Antimicrob. Agents Chemother.* **43**, 406–409 (1999).

12. Eaves, D. J., Liebana, E., Woodward, M. J. & Piddock, L. J. V. Detection of gyrA mutations in quinolone-resistant Salmonella enterica by denaturing high-performance liquid chromatography. *J. Clin. Microbiol.* **40**, 4121–4125 (2002).

13. Baba, T. *et al.* Construction of Escherichia coli K-12 in-frame, single-gene knockout mutants: the Keio collection. *Mol. Syst. Biol.* **2**, 2006.0008 (2006).
