# Supplementary material for: Global pathogenomic analysis identifies known and candidate genetic antimicrobial resistance determinants in twelve species
Source: Nat Commun. 2023 Nov 24;14:7690. doi: 10.1038/s41467-023-43549-9 (PMC10673929; doi:10.1038/s41467-023-43549-9)
Supplement: Supplementary file 3 — Description of Additional Supplementary Files [file 41467_2023_43549_MOESM3_ESM.pdf]

<u>Description of Additional Supplementary Files</u>

Supplementary Data 1 (DatasetS1.json): PATRIC genome IDs for all genomes used.

Supplementary Data 2 (DatasetS2.xlsx): Consolidated SIR phenotypes derived from directly reported SIRs and inference from MICs available on PATRIC. Also includes genome MICs, MIC-SIR mappings used for SIR inference, most common testing standard for each species-drug case determined from PATRIC annotations or manual curation of contributing BioProjects, and distributions of genome MLST subtypes and BioProjects.

Supplementary Data 3 (DatasetS3.xlsx): Distribution of unique AMR genes and cross-species AMR gene analysis. Counts of gene-drug mappings are provided for each species-drug case, along with AMR gene category abbreviations, drug class assignments, curation of AMR gene annotations from PATRIC, and assignments of AMR genes to specific drugs. Species distributions, localization predictions, and function classifications for re-clustered cross-species AMR genes are also included.

Supplementary Data 4 (DatasetS4.xlsx): Distribution of complete *blaTEM* alleles detected and plasmid predictions for contigs containing TEM-116. Also includes MASH distances between all contigs in genomes containing TEM-116 and PLSDB reference plasmids.

Supplementary Data 5 (DatasetS5.xlsx): Summary of SVM model performance and top predictive features across 127 species-drug cases. Includes dataset properties (number of susceptible/resistant genomes, known AMR genes identified), SVM mean test set MCC across 5-fold cross validation, final hyperparameter choices, known AMR genes recovered by either SVM, Pyseer, or Fisher's exact test, and raw lists of top 50 features for each model. Also includes data used in the analysis of individual recovered *gyrA* alleles, comparison between preliminary input feature filters, and GWAS score generalizability analysis.

Supplementary Data 6 (DatasetS6.zip): Sequences associated with the top 50 features from each SVM model. Exact sequences for all such features are provided, as well as the top two most common variants of each type for all sequence clusters related to the features.

Supplementary Data 7 (DatasetS7.xlsx): Filtering results for identifying and categorizing novel AMR gene candidates from SVM models. Also includes abbreviations for drug names.

Supplementary Data 8 (DatasetS8.xlsx): Cell densities achieved by *cycA* and *frdD* mutants under various antibiotic stresses, base media, and supplements. Results of statistical tests between densities achieved for different strains or conditions and predicted *ampC* transcription rates for *frdD* mutants are also included. Also includes all instances of *cycA*, *frdD*, and *ampC* identified for all *E. coli* genomes in this study.
